# Structural basis for the unique ganglioside and cell membrane recognition mechanism of botulinum neurotoxin DC

**DOI:** 10.1038/s41467-017-01534-z

**Published:** 2017-11-21

**Authors:** Sicai Zhang, Ronnie P.-A. Berntsson, William H. Tepp, Liang Tao, Eric A. Johnson, Pål Stenmark, Min Dong

**Affiliations:** 1 Department of Urology, Boston Children’s Hospital, Harvard Medical School, Boston, MA 02115 USA; 2 Department of Microbiology and Immunobiology and Department of Surgery, Harvard Medical School, Boston, MA 02115 USA; 3 Department of Biochemistry and Biophysics, Stockholm University, SE-106 91 Stockholm, Sweden; 4 Department of Medical Biochemistry and Biophysics, Umeå University, SE-901 87 Umeå, Sweden; 5 Department of Bacteriology, University of Wisconsin, Madison, WI 53706 USA

## Abstract

Botulinum neurotoxins (BoNTs), the most potent toxins known, are potential bioterrorism agents. It is well established that all seven serotypes of BoNTs (BoNT/A–G) require complex gangliosides as co-receptors. Here, we report that BoNT/DC, a presumed mosaic toxin between BoNT/D and BoNT/C1, binds and enters efficiently into neurons lacking complex gangliosides and shows no reduction in toxicity in mice deficient in complex gangliosides. The co-crystal structure of BoNT/DC with sialyl-Thomsen-Friedenreich antigen (Sialyl-T) suggests that BoNT/DC recognizes only the sialic acid, but not other moieties in gangliosides. Using liposome flotation assays, we demonstrate that an extended loop in BoNT/DC directly interacts with lipid membranes, and the co-occurring sialic acid binding and loop–membrane interactions mediate the recognition of gangliosides in membranes by BoNT/DC. These findings reveal a unique mechanism for cell membrane recognition and demonstrate that BoNT/DC can use a broad range of sialic acid-containing moieties as co-receptors.

## Abstract (web summary)

Botulinum neurotoxins (BoNTs) are thought to require complex gangliosides, a group of glycosphingolipids, as essential co-receptors to target neurons. Here, the authors show that BoNT/DC represents an exception to this rule and that an extended loop in BoNT/DC penetrates directly into neuronal membranes.

## Introduction

Botulinum neurotoxins (BoNTs) are a family of bacterial toxins produced by diverse strains of anaerobic clostridial bacteria^1, 2^. They cause the disease botulism in humans and animals and are among the six most dangerous potential bioterrorism agents^3^. BoNTs are classified into seven major serotypes (BoNT/A–G)^1, 2^. They are produced as ~150 kDa proteins, composed of a ~50 kDa light chain (LC) and a ~100 kDa heavy chain (HC). The HC contains two functional domains: the translocation domain on the N-terminal half (H_N_) and the receptor-binding domain on the C-terminal half (H_C_). The H_C_ is further composed of two sub-domains (H_CN_ and H_CC_). The LC acts as a protease that cleaves three proteins in neurons: SNAP-25 (the target for BoNT/A, C1, and E), syntaxin 1 (the target for BoNT/C1), and VAMP1/2/3 (vesicle-associated membrane protein, the target for BoNT/B, D, F, and G)^1, 2, 4, 5^. These proteins are members of the SNARE (soluble NSF attachment protein receptor) protein family and form a complex that is essential for synaptic vesicle exocytosis in neurons^6–8^. Cleavage of any one of the three SNARE proteins blocks neurotransmission and causes flaccid paralysis of muscles. Used in minute quantities, BoNTs can attenuate overactive neurons in many medical conditions, making them useful therapeutic toxins^9^.

BoNTs target neurons with exquisite specificity, which is achieved by recognizing two receptors in a “double-receptor” model^5, 10^. The first identified receptor component is a group of glycosphingolipids known as gangliosides^11^. Specific proteins have been also identified as receptors, including synaptic vesicle membrane proteins synaptotagmin I and II (Syt I/II) for BoNT/B, DC, and G, and synaptic vesicle protein 2 (SV2) for BoNT/A, E, and D^12–21^. BoNT/F may also bind to SV2, although whether SV2 is a functional receptor for BoNT/F remains to be established^21–24^. It has also been proposed that BoNT/C1 utilizes two gangliosides, instead of one ganglioside and one protein, as receptors^25, 26^.

**Fig. 1:**
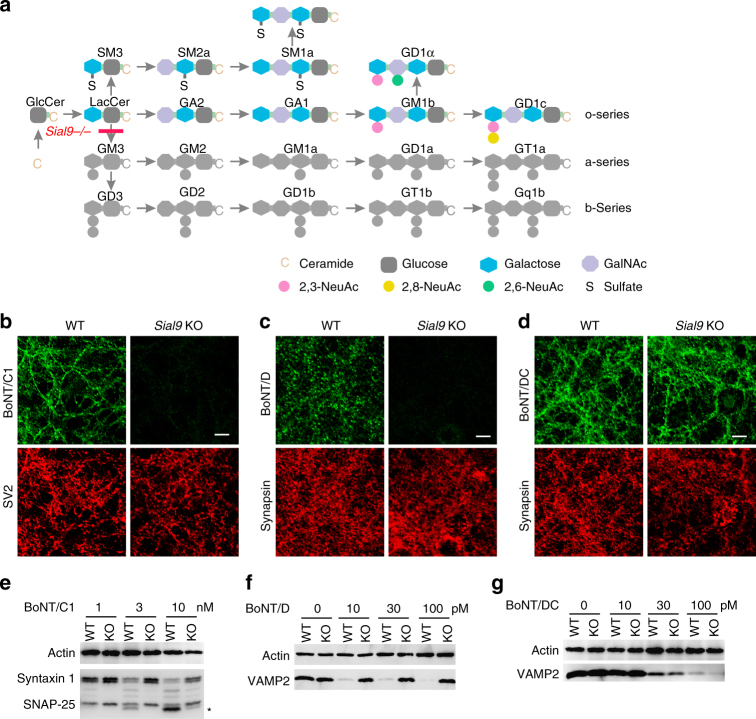
Lacking both a-series and b-series of complex gangliosides reduced binding and entry of BoNT/C1 and D into neurons, but did not affect BoNT/DC. **a** A schematic drawing of ganglioside synthesis pathways, with the step blocked in *Sial9* KO mice marked. **b** WT and *Sial9* KO cortical neurons were exposed to BoNT/C1 (100 nM, 5 min in High-K^+^ buffer). Cells were washed, fixed, and subjected to immunostaining analysis using a polyclonal anti-BoNT/C1 antibody (green). SV2 was co-stained in parallel using a pan-SV2 monoclonal antibody (red) to mark presynaptic terminals. Binding of BoNT/C1 is largely abolished in *Sial9* KO neurons. Scale bar here and thereafter represents 20 µm. **c** Experiments were carried out as described in **b**, except that neurons were exposed to HA-tagged BoNT/D-H_C_ (100 nM). Binding of BoNT/D-H_C_ was detected with a monoclonal HA antibody (green). Synapsin was co-stained to mark presynaptic terminals (red). Binding of BoNT/D-H_C_ is largely abolished in *Sial9* KO neurons. **d** Experiments were carried out as described in **c**, except that neurons were exposed to HA-tagged BoNT/DC-H_C_ (100 nM). BoNT/DC-H_C_ binds equally well to WT and *Sial9* KO neurons. **e** WT and *Sial9* KO neurons were exposed to the indicated concentrations of BoNT/C1 (5 min in High-K^+^ buffer). Neurons were washed and further incubated in toxin-free medium for 8 h. Neurons were harvested and cell lysates were subjected to immunoblot analysis. Cleavage of SNAP-25 by BoNT/C1 generates a slightly smaller fragment, marked with an asterisk. Entry of BoNT/C1 into *Sial9* KO neurons was reduced, as there is less cleavage of SNAP-25 and syntaxin 1. **f**, **g** Experiments were carried out as described in **e**, except that neurons were exposed to BoNT/D (**f**) or BoNT/DC (**g**). Cleavage of VAMP2 by BoNT/D or BoNT/DC results in disappearance of its immunoblot signals. Entry of BoNT/D into *Sial9* KO neurons was blocked, as there was no cleavage of VAMP2 in *Sial9* KO neurons. Entry of BoNT/DC into WT and *Sial9* KO neurons was at similar levels. One of two (**b**–**d**) or three (**e**–**g**) independent experiments is shown

Gangliosides are composed of a hydrophobic ceramide tail and a carbohydrate headgroup. They are the major sialic acid-containing lipids: it has been estimated that ~65% of sialic acids in neuronal plasma membranes are present in gangliosides^27^. The simple form of gangliosides includes GM3 and GD3, which contain a glucose and a galactose (Gal) in their headgroups (Fig. 1a)^28^. The dominant forms of gangliosides in neurons are complex forms including GM1, GD1a, GD1b, and GT1b, which contain an additional N-acetylgalactosamine (GalNAc) and a galactose in their headgroups (GalNAc3-Gal4, Fig. 1a). Several knockout (KO) mouse lines deficient in enzymes required for synthesizing complex gangliosides have been previously utilized to demonstrate that complex gangliosides are an essential co-receptor for all seven BoNTs^20–22, 25, 28–35^. A conserved ganglioside binding site (GBS) with the core residues SXWY has been identified in BoNT/A, B, E, F, and G, as well as in the related tetanus neurotoxin^36–40^. The co-crystal structures of the H_C_ of BoNTs in complex with the headgroup of complex gangliosides have been resolved for BoNT/A, B, and F, showing that the GBS forms multiple contacts with both the sialic acid and the GalNAc3-Gal4 moiety in complex gangliosides^38–40^.

The SXWY motif is not conserved in BoNT/C1 and D. The mechanism by which these two toxins recognize complex gangliosides remains to be fully established. It has been proposed that BoNT/C1 contains two distinct GBSs^25, 26^. One is located near the conserved GBS site; the other one, designated as the Sia-2 site, is adjacent to the protein receptor binding site in BoNT/B. In addition, mutations at an extended loop in BoNT/C1 also reduce toxin binding to gangliosides; this has been designated as a potential ganglioside binding loop (GBL)^25, 41^.

Recent genomic studies revealed multiple subtype and mosaic toxins that are expected to share receptors/substrates with their corresponding prototype toxins^42, 43^. However, BoNT/DC (also known as BoNT/D-SA, D/5995, and D/4947), which is considered a mosaic toxin between BoNT/D and C1, is clearly an exception. The LC and H_N_ of BoNT/DC are almost identical to the corresponding regions in BoNT/D, while its H_C_ is most similar to BoNT/C1-H_C_. However, BoNT/DC but not BoNT/C1 uses Syt I and Syt II as its protein receptors^44, 45^. Thus, BoNT/DC-H_C_ has diverged significantly from BoNT/C1-H_C_. How BoNT/DC recognizes gangliosides remains unknown. Previous studies have attempted to solve the co-crystal structure of BoNT/DC-H_C_ in complex with 3′-sialyllactose, which mimics the headgroup of complex gangliosides, but the electron density of 3′-sialyllactose was too diffuse to resolve any contacts with BoNT/DC-H_C_^46^.

Here, we assessed the requirement of complex gangliosides for BoNT/DC. BoNT/DC was found to be unique among all BoNTs, in that it can efficiently bind and enter neurons lacking complex gangliosides. Utilizing crystal structural studies and liposome flotation assays, we establish that BoNT/DC contains a GBS that recognizes only sialic acids in gangliosides. We further demonstrate that an extended loop in BoNT/DC directly interacts with lipid membranes, and the co-occurring GBS–sialic acid and loop–membrane interactions mediate the binding and recognition of gangliosides in the cell membrane. This mechanism makes BoNT/DC uniquely capable of utilizing a broad range of sialic acid-containing moieties as co-receptors on cell membranes.

## Results

### GM3 synthase KO neurons remain sensitive to BoNT/DC

GM3 is the simplest ganglioside and the precursor for all a-series and b-series of complex gangliosides (Fig. 1a). GM3 is generated by GM3 synthase, encoded by the *Sial9* gene. To determine whether BoNT/DC requires complex gangliosides, we analyzed binding and entry of BoNT/DC into cortical neurons cultured from *Sial9* KO mice. BoNT/C1 and BoNT/D were analyzed in parallel as controls. We first confirmed that binding of BoNT/DC, BoNT/D, and BoNT/C1 on cultured cortical neurons is specific and saturable, as non-tagged H_C_ proteins of these toxins competed with HA-tagged H_C_ proteins and reduced binding/internalization of HA-tagged H_C_ (Supplementary Fig. 1). As expected, neither BoNT/C1 nor BoNT/D was able to bind *Sial9* KO neurons (Fig. 1b, c). In contrast, BoNT/DC-H_C_ binding/internalization levels in wild-type (WT) and Sial9 KO neurons were similar (Fig. 1d).

We further analyzed functional entry of these toxins into *Sial9* KO neurons by examining cleavage of SNARE proteins. Neurons were exposed to toxins and neuronal lysates were subjected to immunoblot assays. As expected, entry of BoNT/C1 into *Sial9* KO neurons was reduced, as cleavage of SNAP-25 and syntaxin 1 was much lower in *Sial9* KO neurons than in WT neurons (Fig. 1e). BoNT/D entry was also blocked in *Sial9* KO neurons, as evidenced by a lack of VAMP2 cleavage (Fig. 1f). In contrast, there were similar degrees of VAMP2 cleavage between WT and *Sial9* KO neurons exposed to BoNT/DC (Fig. 1g). Thus, depleting a-series and b-series of complex gangliosides does not reduce BoNT/DC binding or entry into neurons.

### GM2/GD2 synthase KO neurons remain sensitive to BoNT/DC

**Fig. 2:**
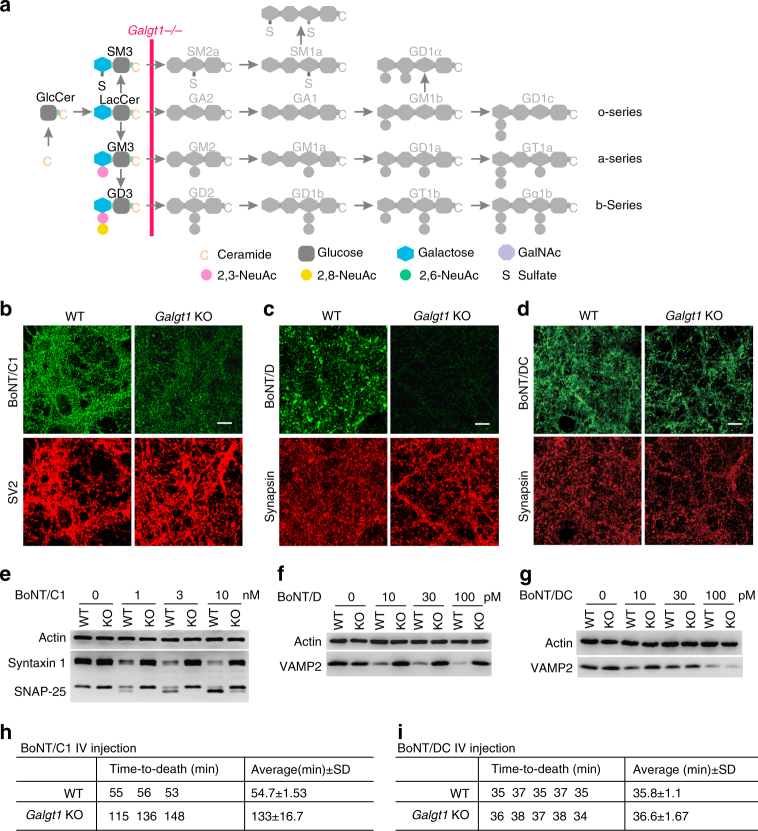
Depleting all complex gangliosides did not affect binding and entry of BoNT/DC into neurons or its toxicity in vivo. **a** The step blocked in *Galgt1* KO mice is marked on ganglioside synthesis pathways. **b**–**d** Experiments were carried out as described in Fig. 1b–d, except with cortical neurons cultured from *Galgt1* KO mice. Binding of BoNT/C1 and BoNT/D-H_C_ was greatly reduced in *Galgt1* KO neurons. In contrast, binding of BoNT/DC-H_C_ to *Galgt1* KO and WT neurons was at similar levels. **e**–**g** Experiments were carried out as in Fig. 1e–g, except with cortical neurons cultured from *Galgt1* KO mice. Entry of BoNT/C1 and BoNT/D into *Galgt1* KO neurons was blocked, while entry of BoNT/DC-H_C_ into *Galgt1* KO and WT neurons was at similar levels. **h** Rapid time-to-death assays were carried out for BoNT/C1. The same amounts of toxins (6 × 10^5^ LD_50_/ml, 100 µl) were injected into WT and *Galgt1* KO mice. Time-to-death of each mouse was recorded. *Galgt1* KO mice survived longer (133 min) than WT mice (55 min), reflecting a reduced sensitivity to BoNT/C1. SD standard deviation. **i** Rapid time-to-death assays were carried out for BoNT/DC as described in **h** (100 µl, ~8 × 10^5^ LD_50_/ml). *Galgt1* KO mice showed survival time similar to WT mice when injected with the same amount of BoNT/DC, suggesting that *Galgt1* KO and WT mice have similar sensitivity to BoNT/DC. One of two (**b**–**d**) or three (**e**–**g**) independent experiments is shown

We next turned to a different KO mouse line lacking GM2/GD2 synthase, which is encoded by the gene *Galgt1* and is required for producing all complex gangliosides (Fig. 2a)^29^. As expected, binding of BoNT/C1 and BoNT/D to *Galgt1* KO neurons was reduced compared to WT neurons (Fig. 2b, c). In contrast, BoNT/DC-H_C_ bound to *Galgt1* KO and WT neurons at similar levels (Fig. 2d). Furthermore, while BoNT/C1 and BoNT/D entry into *Galgt1* KO neurons was blocked (Fig. 2e, f), BoNT/DC entry was not affected, as evidenced by similar degrees of VAMP2 cleavage in *Galgt1* KO and WT neurons (Fig. 2g).

We further utilized a well-established rapid time-to-death assay to examine whether *Galgt1* KO mice are less sensitive to BoNT/DC in vivo compared to WT mice^13, 47^. The assay was carried out by injecting *Galgt1* KO mice and their WT littermates with identical amounts of toxins and then comparing their survival time; longer survival time would indicate lower sensitivity. This rapid time-to-death assay has been successfully utilized to show that *Galgt1* KO mice survived significantly longer than their WT littermates when injected with BoNT/A, B, D, E, or G^15, 20, 21, 32^, demonstrating that it is capable of detecting the difference in sensitivity to BoNTs in vivo. Using this assay, we first tested BoNT/C1 as a control. As expected, *Galgt1* KO mice survived much longer than WT mice when injected with the same amount of BoNT/C1 (Fig. 2h). In contrast, *Galgt1* KO mice survived for about as long as WT mice when injected with the same amount of BoNT/DC (Fig. 2i), suggesting that complex gangliosides are not required for BoNT/DC toxicity in vivo. As all seven BoNTs need complex ganglioside as essential co-receptors, BoNT/DC is clearly an outlier.

### Depleting gangliosides reduces binding and entry of BoNT/DC

**Fig. 3:**
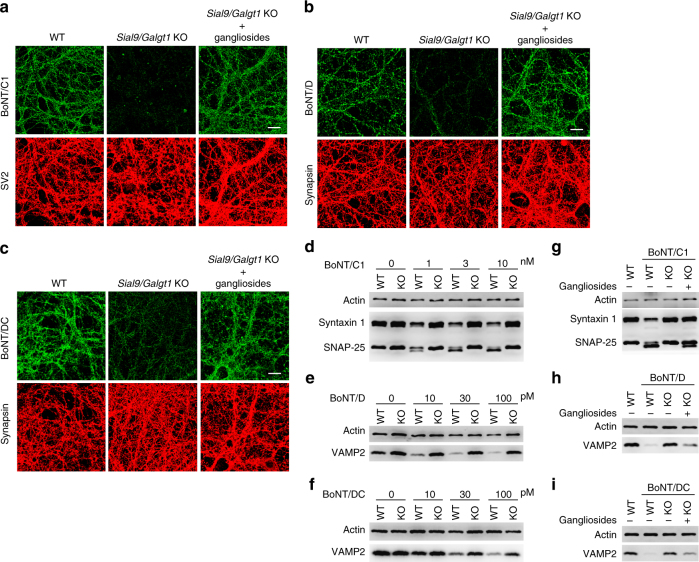
Depleting all gangliosides in *Sial9/Galgt1* double-KO neurons reduced binding and entry of BoNT/DC. **a**–**c** Binding of BoNT/C1 (**a**), BoNT/D-H_C_ (**b**), and BoNT/DC-H_C_ (**c**) to WT, *Sial9/Galgt1* double-KO, and *Sial9/Galgt1* double-KO neurons pre-loaded with exogenous gangliosides were examined by immunofluorescence staining, under the same assay conditions described in Fig. 1b–d. Binding of BoNT/C1 and BoNT/D-H_C_ was abolished in *Sial9/Galgt1* double-KO neurons. Binding of BoNT/DC-H_C_ was reduced in *Sial9/Galgt1* double-KO neurons. Exogenous gangliosides restored binding of all three toxins to *Sial9/Galgt1* double-KO neurons. **d**–**f** WT and *Sial9/Galgt1* double-KO neurons were exposed to BoNT/C1 (**d**), BoNT/D (**e**), or BoNT/DC (**f**) at the indicated concentrations. Cell lysates were analyzed by immunoblot as described in Fig. 1e–g. Entry of BoNT/C1 and BoNT/D into *Sial9/Galgt1* double-KO neurons was largely blocked, as there was a lack of cleavage of SNAP-25 and syntaxin 1 (**d**), and VAMP2 (**e**). BoNT/DC still entered *Sial9/Galgt1* double-KO neurons and cleaved VAMP2, but the levels of VAMP2 cleavage were lower in *Sial9/Galgt1* double-KO neurons than in WT neurons (**f**), indicating that entry of BoNT/DC was reduced in *Sial9/Galgt1* double-KO neurons compared to WT neurons. **g**–**i** Pre-loading exogenous gangliosides onto *Sial9/Galgt1* double-KO neurons restored functional entry of BoNT/C1 (10 nM, **g**), BoNT/D (100 pM, **h**), and BoNT/DC (100 pM, **i**), analyzed by immunoblot as described in Fig. 1e–g. One of two (**a**–**c**) or three (**d**–**i**) independent experiments is shown

*Galgt1* KO mice still express simple gangliosides GM3 and GD3 (Fig. 2a). By breeding *Galgt1* KO mice with *Sial9* KO mice, we generated double-KO mice that do not express any gangliosides. As gangliosides are essential, these double-KO mice die within 24 h after birth, but cortical neurons can be cultured from newborn double-KO pups. As expected, both BoNT/C1 and BoNT/D failed to bind double-KO neurons (Fig. 3a, b). Binding was restored when exogenous mixed brain gangliosides were pre-loaded onto neuronal membranes (Fig. 3a, b), confirming that the defect is due to a lack of gangliosides. Similarly, binding of BoNT/DC-H_C_ was reduced in neurons cultured from *Galgt1/Sial9* double-KO mice and was restored by loading exogenous gangliosides onto neuronal membranes (Fig. 3c), suggesting that BoNT/DC recognizes a moiety in gangliosides.

We further examined functional entry of these three toxins into *Galgt1/Sial9* double-KO neurons. As expected, entry of BoNT/C1 and D was largely blocked (Fig. 3d, e). Entry of BoNT/DC was lower in *Galgt1/Sial9* double-KO neurons compared to WT neurons (Fig. 3f), but not completely blocked, as evidenced by cleavage of VAMP2 in *Galgt1/Sial9* double-KO neurons when exposed to BoNT/DC (Fig. 3f). Pre-loading exogenous gangliosides restored entry of BoNT/C1, D, and DC (Fig. 3g–i). These results suggest that BoNT/DC recognizes a moiety that is abundant in gangliosides, but may still be present at lower levels on neuronal surfaces in the absence of all gangliosides.

### Co-crystal structure of BoNT/DC in complex with Sialyl-T

Sialyl-T consists of a sialic acid (Sia), a galactose (Gal), and an N-acetylglucosamine (GalNAc), mimicking the structure in the headgroup of major brain complex gangliosides such as GD1a and GT1b (Fig. 1a). Previous structural studies have demonstrated that the three moieties (Sia5, Gal4, and GalNAc3) found in Sialyl-T are responsible for interactions between complex gangliosides and the GBS in BoNT/A, B, and F^38–40^. To identify the potential moiety on gangliosides recognized by BoNT/DC, we sought to determine the X-ray co-crystal structure of BoNT/DC-H_C_ in complex with Sialyl-T.

**Fig. 4:**
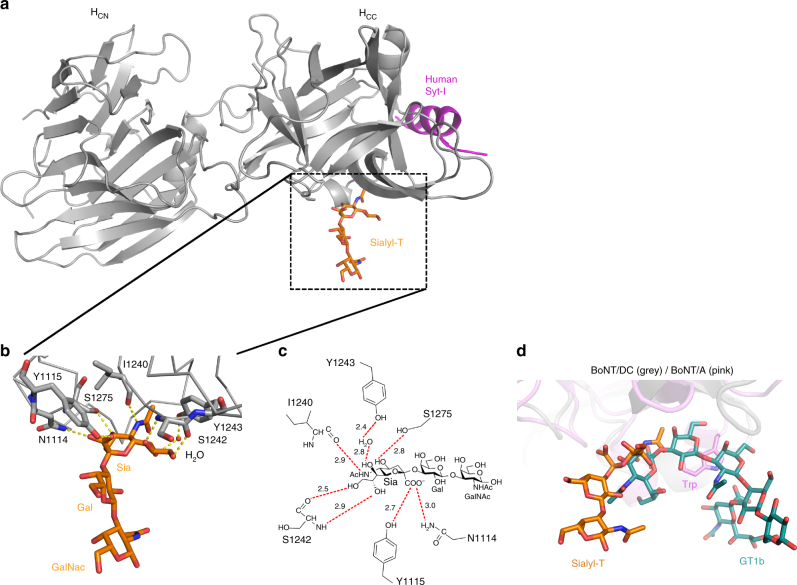
Co-crystal structure of BoNT/DC-H_C_ in complex with Sialyl-T. **a** Overall view of BoNT/DC (gray) with the bound Sialyl-T molecule (orange). Human Syt I (magenta) is modeled in from the previously solved structure of BoNT/DC–Syt I complex (PDB code: 4ISQ). **b** Close-up view of the Sialyl-T binding site of BoNT/DC, with the protein shown in ribbon, Sialyl-T and the interacting residues as sticks, and one interacting water as a red sphere. Possible hydrogen bonds are shown as dashed lines. **c** Schematic representation of the possible hydrogen bonds between Sialyl-T and BoNT/DC (red dashed lines). The distance for each bond is shown in Å. **d** Structural comparison between BoNT/A and BoNT/DC on ganglioside binding. The Sialyl-T binding site in BoNT/DC (shown in gray) is located at a position similar to the GBS in BoNT/A (shown in pink). The sialic acid (Sia) of Sialyl-T (shown in orange) sits in a similar position as the Sia5 group of the GT1b (shown in cyan) bound to BoNT/A. The rest of the Sialyl-T carbohydrates are at a different position than the Gal4-GalNAc3 moieties in GT1b, which form both hydrogen bonds and stacking interactions to a Trp residue in the GBS of BoNT/A

Co-crystallization of BoNT/DC-H_C_ with Sialyl-T yielded crystals diffracting to 2.6 Å, with space group P4_1_2_1_2 (Table 1, Supplementary Fig. 2). The overall structure of BoNT/DC-H_C_ was identical to the previously reported structure of BoNT/DC-H_C_ in complex with its protein receptors Syt I/II^45^ (Fig. 4a). The complex contains two molecules per asymmetric unit (ASU), with well-defined electron density for Sialyl-T. Due to crystal contacts, Sialyl-T bound to chain A in the crystal is highly ordered, whereas the other Sialyl-T bound to chain B has much weaker electron density in the parts of Sialyl-T not interacting with BoNT/DC-H_C_. No significant differences were observed in the interactions between Sialyl-T and BoNT/DC-H_C_ between the two Sialyl-T molecules, so from here on all data we report are based on the well-defined Sialyl-T molecule bound to chain A. The structure revealed that Sialyl-T binds to BoNT/DC-H_C_ with a total of seven hydrogen bonds, all originating from the terminal sialic acid (Fig. 4b, c). There are two hydrogen bonds to S1242, one each to N1114, Y1115, I1240, and S1275. An additional hydrogen bond is formed with Y1243 via a bridging water molecule (Fig. 4c). Together, these contacts form a specific binding site for the terminal sialic acid, while the Gal-GalNAc backbone in Sialyl-T does not contribute any interactions with BoNT/DC-H_C_.

**Table 1:** Data collection and refinement statistics

Data collection	BoNT/DC-H_C_ + Sialyl-T
Space group	P4_1_2_1_2
*Cell dimensions*	
*a*, *b*, *c* (Å)	109.7, 109.7, 210.5
*α*, *β*, *γ* (°)	90, 90, 90
Resolution (Å)	48.6–2.6 (2.69–2.6)
*R*_merge_	0.23 (1.39)
*I*/*σ* (*I*)	13.2 (2.5)
Completeness (%)	99.2 (92.6)
CC(1/2)*	0.99 (0.79)
Redundancy	14.5
*Refinement*	
Resolution	48.6–2.6
No. of unique reflections	38,598
*R*_work_/*R*_free_	0.20/0.23
*No. of atoms*	
Protein	6709
Sialyl-T	92
Water	143
*B-factors*	
Protein	41
Sialyl-T	66
Water	33
*R.m.s. deviations*	
Bond lengths (Å)	0.006
Bond angles (°)	1.18

Values in parenthesis are for the highest resolution shell

The location of the Sialyl-T binding site in BoNT/DC is homologous to the well-defined GBS site in other BoNTs (Fig. 4d). The major difference is that this site in BoNT/DC forms contacts with only the sialic acid, but not the Gal-GalNAc, whereas the conserved GBS site in other BoNTs interacts with all three moieties. For instance, the Gal4 in gangliosides forms crucial aromatic stacking interactions with a tryptophan residue conserved in the GBS of BoNT/A, B, and F^38–40^. This tryptophan residue is not conserved in BoNT/DC. Essentially, the GBS in BoNT/DC has “degenerated” into a sialic acid-binding site. Thus, the reason BoNT/DC showed no defects in binding or entry into complex-ganglioside-deficient neurons could be that these neurons still express simple gangliosides (likely at elevated levels) that contain sialic acids.

### Binding of DC-H_C_ to gangliosides in liposome flotation assay

**Fig. 5:**
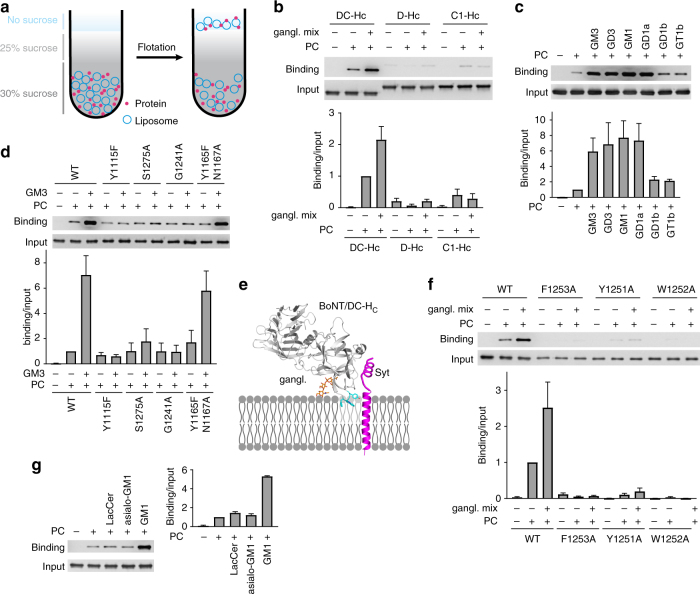
Binding of BoNT/DC-H_C_ to ganglioside-containing liposomes. **a** A schematic drawing of the liposome flotation assay. **b** Experiments were carried out as depicted in **a**. Liposomes containing PC alone or PC plus a brain ganglioside mixture (gangl. mix, 1%) were incubated with HA-tagged BoNT/DC-H_C_, BoNT/D-H_C_, or BoNT/C1-H_C_. Samples were centrifuged for 1 h at 240,000 × *g* in a sucrose gradient. Liposome fractions that floated to the top of the gradient were collected and subjected to immunoblot analysis. Samples without liposomes served as a negative control. BoNT/DC-H_C_ showed a basal level of binding to PC liposomes. Adding gangliosides increased binding of BoNT/DC-H_C_ to liposomes. BoNT/D-H_C_ and BoNT/C1-H_C_ did not show detectable binding to liposomes under the same assay conditions. **c** Experiments were carried out as described in **b**, except with indicated individual ganglioside species instead of a mixture of brain gangliosides. Both simple gangliosides (GM3 and GD3) and complex gangliosides (GM1 and GD1a) enhanced binding of BoNT/DC-H_C_ to liposomes. GD1b and GT1b also increased binding of BoNT/DC-H_C_, but to a much lesser degree than GM1 and GD1a. **d** Experiments were carried out as described in **b**, with the indicated mutants of BoNT/DC-H_C_ and GM3-loaded liposomes. Mutations at the GBS (Y1115F, S1275A, and G1241A) abolished ganglioside-mediated binding to liposomes, as they showed similar levels of binding in PC alone and GM3-containing liposomes. In contrast, Y1165F/N1167A did not affect ganglioside-mediated binding to liposomes. None of these mutations reduced binding of BoNT/DC-H_C_ to PC liposomes compared to WT. **e** Model of BoNT/DC-H_C_ on plasma membranes. BoNT/DC is anchored onto plasma membranes by both Syt-binding and GBS–sialic acid interactions, putting the protein at an ideal angle to allow the extended loop (blue) to penetrate into the lipid membrane. **f** Experiments were carried out as described in **b**. Mutating any one of the three residues at the tip of the extended loop (F1253A, Y1251A, or W1252A) abolished binding of BoNT/DC-H_C_ to PC liposomes. Adding gangliosides did not rescue binding. **g** Experiments were carried out as described in **b**. Binding of BoNT/DC-H_C_ to liposomes containing LacCer or asialo-GM1 was at similar levels as to PC liposomes, while GM1 increased binding of BoNT/DC-H_C_. One of three (**b**–**d**, **f**, **g**) independent experiments is shown. Immunoblot was quantified using ImageJ (*n* = 3) and normalized to the levels of BoNT/DC-H_C_ binding to PC liposomes in all panels. Error bars represent standard deviation

We next utilized the liposome flotation assay to further characterize BoNT/DC binding to gangliosides using well-defined minimal components. The assay was carried out by first incubating BoNT/DC-H_C_ with liposomes containing either phosphatidylcholine (PC) alone or PC plus gangliosides (1%). The mixture was then subjected to centrifugation in a sucrose gradient (Fig. 5a). Liposomes float to the top of the gradient due to their low density; proteins bound to the liposomes float together with liposomes, whereas unbound proteins remain at the bottom. As shown in Fig. 5b, BoNT/DC-H_C_ showed a low level of binding to PC liposomes. Adding a mixture of brain gangliosides into liposomes increased BoNT/DC-H_C_ binding. We note that detection of BoNT/DC-H_C_ binding to liposomes requires relatively high concentrations of BoNT/DC-H_C_ (1 µM), suggesting that the binding affinity between BoNT/DC-H_C_ and liposomes is weak. We also examined HA-tagged BoNT/D-H_C_ and BoNT/C1-H_C_ in parallel, but their interactions with liposomes are too weak to be reliably detected under our assay conditions (Fig. 5b).

Using this assay, we found that BoNT/DC-H_C_ showed similar levels of binding to liposomes containing GM3, GD3, GM1, or GD1a, confirming that BoNT/DC can bind to both simple and complex gangliosides (Fig. 5c). Binding of BoNT/DC-H_C_ to liposomes containing GD1b and GT1b was considerably less than to liposomes containing GM1 and GD1a (Fig. 5c), though the reason for this difference among complex gangliosides remains unclear.

We next evaluated whether ganglioside binding is mediated by the GBS identified in our crystal structure. Among six residues that form direct bonds to the sialic acid, four have been previously tested by Neumket et al. in mutagenesis screening, which showed that N1114A, I1240A, S1242A, and Y1243A all abolished BoNT/DC-H_C_ binding to immobilized gangliosides and to cell surfaces^46^. These results are consistent with the findings of our crystal structure. To further validate the structural data, we created additional point mutations at the remaining two residues that form direct contacts with the sialic acid: Y1115F and S1275A. We also tested G1241A: our structure predicts that it would create a steric clash to block binding of sialic acids. In addition, we tested a double mutation Y1165F/N1167A. These two mutations are located at the site homologous to the Sia-2 previously proposed in BoNT/C1^25, 26^ (Supplementary Fig. 3), and this double mutation was designed to probe whether BoNT/DC contains the same Sia-2 site as BoNT/C1. All these point mutations can still bind to the protein receptor Syt II (Supplementary Fig. 4), confirming that they are properly folded. As shown in Fig. 5d, all three point mutations (Y1115A, S1275A, and G1241A) at the GBS abolished ganglioside-mediated binding to liposomes, as they showed similar levels of binding in ganglioside-containing liposomes and PC liposomes. In contrast, the Y1165F/N1167A mutant was similar to WT and showed increased binding to ganglioside-containing liposomes compared to PC liposomes (Fig. 5d), suggesting that BoNT/DC does not contain the Sia-2 site. Consistently, WT and Y1165F/N1167A mutant showed similar levels of apparent dissociation constants (*K*_D_) toward biotinylated GM1 measured by the biolayer interferometry assay (Supplementary Fig. 5), confirming that Y1167F/N1167A mutations did not affect binding of BoNT/DC-H_C_ to gangliosides.

### Direct binding of BoNT/DC to PC liposomes

We next examined the ganglioside-independent binding of BoNT/DC to PC liposomes (Fig. 5b). Mutations at the GBS or the Sia-2 site in BoNT/DC did not affect its binding to PC liposomes, suggesting that it is not mediated by these two sites (Fig. 5d). To explore the potential mechanism for this binding to PC liposomes, we modeled BoNT/DC-H_C_ onto the plasma membrane based on the co-crystal structures of BoNT/DC-H_C_ in complex with Syt and Sialyl-T. The model revealed that an extended loop between the GBS and the Syt binding site of BoNT/DC is located at an ideal position to penetrate into the plasma membrane (blue color, Fig. 5e). This loop has been previously proposed to be a GBL, as mutations within it abolished binding of BoNT/DC-H_C_ to immobilized gangliosides^25, 41^. Furthermore, Neumket et al. also confirmed the importance of this loop and showed that point mutations (Y1251A, W1252A, or F1253A) at the tip of this loop or deletion of the loop altogether abolished binding of BoNT/DC-H_C_ to gangliosides and to the surface of P19 cells^46^. However, whether this loop specifically recognizes gangliosides has not been established. As the ceramide tail of gangliosides forms hydrophobic membranes, an alternative possibility is that this loop contributes to ganglioside binding via non-specific penetration into the hydrophobic interior of the membrane, as suggested by our modeling (Fig. 5e). This non-specific loop–membrane interaction may mediate BoNT/DC binding to PC liposomes. To test this idea, we examined whether the three point mutations previously reported by Neumket et al. (Y1251A, W1252A, and F1253A) affect BoNT/DC-H_C_ binding to PC liposomes. As shown in Fig. 5f, each of the three point mutations abolished binding of BoNT/DC-H_C_ to PC liposomes under our assay conditions, indicating that all three tip residues are critical for ganglioside-independent toxin–liposome interactions. Adding gangliosides into PC liposomes did not rescue binding of Y1251A, W1252A, or F1253A mutants (Fig. 5f), suggesting that the GBS–sialic acid interaction alone is not sufficient to mediate robust binding to ganglioside-containing liposomes. It is likely that a combination of GBS–sialic acid and loop–membrane interactions is required. This dual requirement may explain previous findings that mutations in the loop region alone abolished binding of BoNT/DC to gangliosides^25, 41, 46^.

To further determine whether the extended loop in BoNT/DC has any preference for gangliosides over other lipid membranes, we examined binding of BoNT/DC-H_C_ to liposomes containing LacCeramid (LacCer), which is the precursor of GM3 and lacks only GM3’s sialic acid (Fig. 1a), as well as liposomes containing asialo-GM1, which lacks only GM1’s sialic acid. BoNT/DC-H_C_ bound to liposomes containing LacCer or asialo-GM1 and PC liposomes at similar levels (Fig. 5g), suggesting a lack of specific recognition of either the headgroup or ceramide tail of asialo-gangliosides by the extended loop in BoNT/DC. The presence of GM1 in liposomes increased binding of BoNT/DC-H_C_, confirming that robust binding of BoNT/DC to liposomes requires both GBS–sialic acid and loop–membrane interactions.

### BoNT/DC binding to neurons

**Fig. 6:**
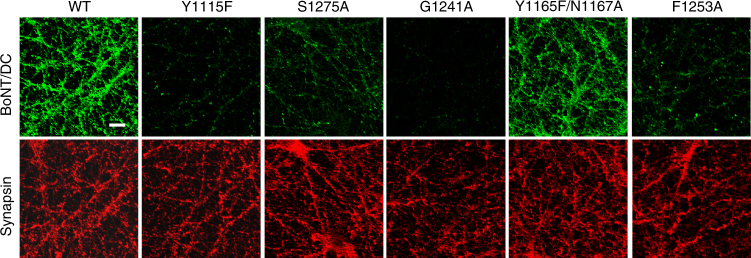
Mutations at the GBS or at the extended loop reduced binding of BoNT/DC-H_C_ to neurons. Experiments were carried out as described in Fig. 1d, with WT or the indicated mutants of BoNT/DC-H_C_. Mutations at the GBS (Y1115F, S1275A, and G1241A) and a mutation at the extended loop (F1253A) all reduced binding of BoNT/DC-H_C_ to neurons. Y1165F/N1167A did not affect binding BoNT/DC-H_C_ to neurons. One of two independent experiments is shown

We next assessed the contribution of the GBS and the extended loop for BoNT/DC binding to neuronal surfaces. As shown in Fig. 6, mutations at the GBS, including Y1115F, S1275A, and G1241A, all reduced binding of BoNT/DC-H_C_ to cultured neurons. The mutation at the extended loop (F1253A) also largely abolished binding of BoNT/DC-H_C_ to neurons (Fig. 6). These results further support a model in which robust binding of BoNT/DC to neuronal surfaces requires a combination of GBS–sialic acid and loop–membrane interactions. The Y1165F/N1167A mutant binds well to neurons, suggesting that this location does not contribute to BoNT/DC binding to neurons.

### GBS–sialic acid interactions mediate BoNT/DC binding to SV2

The finding that the GBS of BoNT/DC-H_C_ binds only to the sialic acid in gangliosides reminded us of a previous observation: BoNT/DC-H_C_ always pulls down significant amounts of SV2 from brain lysates, in addition to its protein receptors Syt I/II^44^. SV2 is clearly not an essential component of BoNT/DC receptors, as BoNT/DC entered neurons lacking SV2 and neurons that still express SV2 at similar levels (Supplementary Fig. 6). SV2 is known to form a complex with Syt I/II, which may explain some level of SV2 pulldown^48^. However, BoNT/B pulls down more Syt I/II, but less SV2 than BoNT/DC in the same assay^44^.

**Fig. 7:**
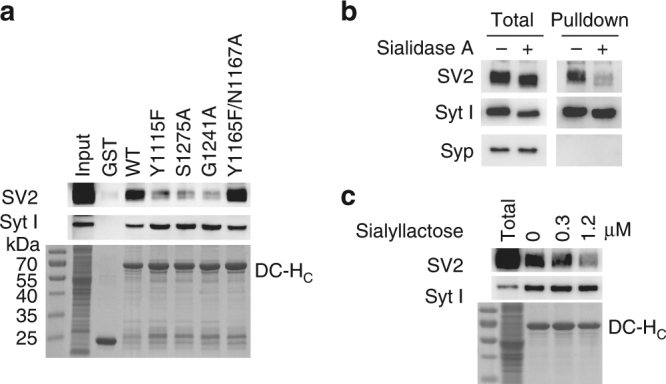
Pulldown of SV2 by BoNT/DC from brain lysates was mediated by binding of the GBS of BoNT/DC to sialic acids in SV2. **a** GST or GST-fused BoNT/DC-H_C_ were immobilized on beads and incubated with rat brain detergent lysates (in Triton X-100). Pellets were subjected to immunoblot analysis detecting SV2 (upper panel) and Syt I (middle panel), or subjected to Coomassie Blue staining to show GST and GST-fused BoNT/DC-H_C_ proteins (lower panel). WT BoNT/DC-H_C_ pulled down Syt I and SV2. Mutations at the GBS (Y1115F, S1275A, and G1241A) did not affect Syt I pulldown, but reduced SV2 pulldown. Y1165F/N1167A behaved similarly to WT BoNT/DC-H_C_. **b** Pull-down assays were carried out using GST-fused BoNT/DC-H_C_ and rat brain detergent extracts pre-treated with sialidase A (0.0125 U per 100 µl lysates for 1 h at 37 °C). Pellets were subjected to immunoblot analysis detecting SV2, Syt I, and Syp. Sialidase treatment cleaves terminal sialic acids from glycosylated proteins, thus reducing the molecular weights of SV2, Syt I, and Syp. Sialidase treatment did not affect Syt I pulldown, but reduced SV2 pulldown. Syp served as a negative control, which did not interact with BoNT/DC-H_C_. **c** Pull-down assays were carried out as described in **a**, with rat brain detergent extracts in the presence of indicated concentrations of sialyllactose. Sialyllactose did not affect Syt I pulldown, but reduced SV2 pulldown in a dose-dependent manner. One of three (**a**–**c**) independent experiments is shown

SV2 is heavily glycosylated in neurons. As glycosylated SV2 contains abundant sialic acids, we sought to determine whether BoNT/DC interacts directly with SV2 via GBS–sialic acid interactions. As shown in Fig. 7a, mutations at the GBS (Y1115F, S1275A, and G1241A) did not affect pulldown of Syt I from brain lysates by BoNT/DC-H_C_, but reduced pulldown of SV2. Mutations at the Sia-2 site (Y1165F/N1167A) still pulled down similar amounts of SV2 as WT BoNT/DC, serving as a control. Furthermore, pre-treating brain lysates with sialidase A, which cleaves off the terminal sialic acids, reduced pulldown of SV2 by BoNT/DC (Fig. 7b). Sialidase A treatment did not affect Syt I pulldown as expected. Finally, adding sialyllactose as a competitor reduced SV2 pulldown from brain lysates by BoNT/DC, but did not affect Syt I pulldown (Fig. 7c). Together, these data demonstrate that pulldown of SV2 by BoNT/DC requires binding of the GBS to sialic acids in glycosylated SV2. Interestingly, BoNT/DC did not pulldown another glycosylated protein, synaptophysin (Syp, Fig. 7b), suggesting that GBS–sialic acid interaction alone is not enough to pulldown glycosylated proteins. Formation of the SV2–Syt–BoNT/DC triple complex may provide an additional force for BoNT/DC to pulldown SV2 in brain lysates.

## Discussion

The role of complex gangliosides as essential co-receptors is well-established dogma for the BoNT family of toxins. Here, we found that BoNT/DC is distinct from all known BoNTs: it contains a GBS that recognizes only sialic acids, with no direct contact with the rest of the carbohydrate headgroup of complex gangliosides. In essence, the GBS in BoNT/DC becomes a sialic acid-binding site. This gives BoNT/DC the unique ability to utilize a broad range of sialic acid-containing molecules as co-receptors.

We further found that the GBS–sialic acid interaction alone is not sufficient to mediate robust binding of BoNT/DC to either ganglioside-containing liposomes or neuronal surfaces, as mutations at an extended loop in BoNT/DC abolishes binding of BoNT/DC to liposomes and neurons. Previous studies have proposed that this loop in BoNT/DC and the homologous loop in BoNT/C1 act as GBLs^25, 41^. However, there is a lack of evidence for any specific interactions between this loop and gangliosides. An alternative explanation is that this loop interacts with lipid membranes via hydrophobic interactions, as residues at the tip of this loop are ideally positioned to penetrate into membranes. Such a role has been previously proposed for the homologous loops in BoNT/B and BoNT/C1, but has not been examined experimentally^17, 25, 41^. Utilizing the liposome flotation assay, we were able to analyze the direct and ganglioside-independent binding of BoNT/DC to liposomes and demonstrate that point mutations at the tip of the extended loop abolish toxin binding to liposomes, thus suggesting that the extended loop interacts with lipid membranes independent of gangliosides.

The loop–membrane interaction not only enhances the overall binding avidity to neuronal surfaces, but may also provide a degree of specificity toward gangliosides by imposing a spatial constraint, so that the GBS in BoNT/DC can sample sialic acids only within a certain distance from the neuronal membrane. Indeed, depleting all gangliosides in neurons greatly diminished binding and entry of BoNT/DC, suggesting that BoNT/DC still utilizes gangliosides as its primary co-receptors in neurons. Furthermore, binding of BoNT/DC to its protein receptors, Syt I/II, also anchors BoNT/DC onto membranes in a spatially conserved manner^45^, which might further enhance the preference of BoNT/DC toward the sialic acids in gangliosides on neuronal surfaces.

In essence, BoNT/DC recognizes neuronal membranes by combining the recognition of the two most common moieties on cell surfaces: sialic acids and lipid membranes. Each interaction alone is not sufficient to fix the toxin onto the cell membrane. Only when the two interactions combine, can the toxin be anchored onto the cell membrane. The specificity of BoNT/DC toward neurons is further ensured by its binding to protein receptors Syt I/II, which become exposed to the neuronal surface only transiently during vesicle exocytosis.

We have previously shown that BoNT/DC pulls down significant amounts of SV2 from brain lysates, in addition to Syt I/II^44^. Here, we conclude that pulldown of SV2 requires BoNT/DC binding to sialic acids in glycosylated SV2. It has been clearly shown that binding and entry of BoNT/DC are not affected in neurons lacking SV2^44^, thus GBS–SV2 interactions are likely irrelevant in normal neurons. However, BoNT/DC might be able to utilize sialic acids on glycosylated proteins in the absence of all gangliosides, which may explain entry of BoNT/DC into *Galgt1/Sial9* double-KO neurons.

BoNT/DC is the only BoNT that breaks the strict reliance on complex gangliosides. Its unique recognition strategy of combining loop–membrane interactions with sialic acid binding may give this toxin the advantage of utilizing a broad range of sialic acid-containing molecules as co-receptors. Simple gangliosides such as GM3 are widely expressed in non-neuronal cells. The unique ganglioside recognition strategy may provide a lesson in engineering toxins to target a wide range of cells.

## Methods

### Materials and toxins

Mouse monoclonal antibodies for VAMP2 (Cl69.1), SNAP-25 (Cl71.2), syntaxin 1 (HPC-1), Syp (Cl7.2), Syt I (Cl41.1), and SV2 (pan-SV2) were generously provided by E. Chapman (Madison, WI) and are available from Synaptic Systems (Goettingen, Germany). Rabbit polyclonal antibody against BoNT/C1 was generated in E. Johnson’s lab. The following antibodies were purchased from the indicated vendors: mouse monoclonal anti-HA (16B12, Covance), rabbit polyclonal anti-synapsin (Millipore), and mouse monoclonal anti-actin (Sigma). All gangliosides and LacCer were purchased from Matreya LLC (Pleasant Gap, PA). BoNT/C1 (Brazil), BoNT/DC (strain 5995), and BoNT/D (strain 1873) were purified in E. Johnson’s lab. PC lipids were purchased from Avanti (Alabaster, AL). Sialidase A was purchased from Prozyme (Hayward, CA). Sialy-T and Sialyllactose were purchased from Carbosynth.

### Mouse lines

The *Sial9* KO mouse line was obtained from R.L. Proia (NIH)^30^. The *Galgt1* KO mouse line was obtained from the Consortium for Functional Glycomics and has been previously described^29, 49^. SV2A/SV2B double-KO mice were obtained from Jackson Laboratory and have been previously described^21^. All procedures were conducted in accordance with the guidelines approved by the Institute Animal Care and Use Committee (IACUC) at the University of Wisconsin—Madison and the IACUC at Boston Children’s Hospital.

### Rapid time-to-death assay in vivo

Rapid time-to-death assay was carried out as we described previously^13, 47^. Briefly, the same amount of BoNT/C1 was injected into WT and *Galgt* 1 KO mice intravenously (100 µl, 6 × 10^5^ LD_50_/ml, lateral tail vein) and survival time recorded. BoNT/DC was analyzed following the same procedure. We note that BoNT/DC dose was estimated using BoNT/A standard curve in the rapid time-to-death assay. Both male and female mice were used randomly and experiments were not done blindly.

### Constructs and protein purifications

The cDNA encoding the H_C_ of BoNT/DC (residues 859–1285, GenBank: AB461915.1), the H_C_ of BoNT/D (residues 859–1276, GenBank: CAA38175.1), and the Hc of BoNT/C1 (residues 867–1291, GenBank: CAA51313.1) were synthesized by GenScript Inc. (New Brunswick, NJ) with codon optimized for *E. coli* expression. They were sub-cloned into both regular pET28a vector and a modified pET28a vector with an N-terminal HA tag. Mouse Syt II (residues 40–63) was subcloned into pGEX4T-1 to generate Glutathione S-transferase (GST)-fused Syt II. Protein purification was carried out as previously described^21^. Point mutations in BoNT/DC-H_C_ were generated using the QuikChange mutagenesis kit (Agilent Technologies) and verified by sequencing.

### Neuron culture, toxin binding, and entry assays

Rat cortical neurons were prepared from E18-19 embryos. Dissected hippocampi were dissociated with papain following manufacture instructions (Worthington Biochemical, NJ). Cells were plated on poly-D-lysine-coated glass coverslips and cultured in Neurobasal medium supplemented with B-27 (2%) and Glutamax (Invitrogen) as we described previously^21^. Cortical neurons cultured from KO mouse lines were prepared from newborn pups. Toxin binding and entry into cortical neurons was carried out in high-K^+^ buffer (the same as PBS, but adjusted to 56 mM KCl and 87 mM NaCl plus 1 mM CaCl_2_) for 5 min as previously described^21^. For binding assays, cells were fixed and permeabilized for immunostaining analysis. We note that both surface-bound and internalized toxins are detected, and we used “binding” as a general term representing both bound and internalized toxins in this assay. Briefly, cells were washed three times. Immunostaining was carried out by fixing cells with 4% paraformaldehyde, permeabilized with 0.3% Triton in PBS solution, and incubated with primary antibodies for 1 h at room temperature (RT), followed by the incubation with secondary antibodies conjugated with Alexa dye for 1 h at RT. Fluorescence images were collected using a Leica TCS SP8 confocal microscope with a 40× objective. For functional entry assays, cells were subsequently cultured for 8 h after exposure to toxins. Cell lysates were collected and subjected to immunoblot analysis.

### Pull-down assay and immunoblot analysis

Rat brain detergent extracts were prepared by homogenizing one fresh dissected adult rat brain in 15 ml 320 mM sucrose buffer, followed by a centrifugation at 3000 × *g* for 2 min at 4 °C. Supernatants were centrifuged at 15,000 × *g* for 12 min. The pellet was collected and solubilized for 30 min in 15 ml Tris-buffered saline (TBS: 20 mM Tris, 150 mM NaCl) plus 2% of Triton X-100 and a cocktail of protease inhibitors (Roche, CA). Samples were centrifuged at 35,000 × *g* for 20 min to remove the insoluble materials. The final brain detergent extracts yielded ~2 mg per ml proteins. GST pull-down assays with brain detergent lysates were carried out using GST fusion proteins or GST protein immobilized on Glutathione-Sepharose beads (GE Bioscience). Twenty µg of GST-BoNT/DC-H_C_ WT or indicated mutants were incubated with 0.5 ml rat brain detergent extracts for 1 h at 4 °C. Pull-down assays with recombinant proteins were carried out using 20 µg of indicated GST-Syt II fragments or GST, incubated with BoNT/DC-H_C_ (20 µg) in 200 µl TBS buffer plus 0.5% Triton X-100 for 1 h at 4 °C. Beads were washed three times using Tris-buffered saline plus 0.5% Triton X-100. Ten percent of bound materials were subjected to SDS-PAGE followed by either Coomassie Blue staining or immunoblot analysis using enhanced chemiluminescence method (Pierce).

### Biolayer interferometry assay

The binding affinities between BoNT/DC-H_C_ and biotinylated GM1 were characterized using the Blitz system (ForteBio). Briefly, biotinylated GM1 was loaded onto streptavidin-conjugated sensors by incubating the sensors in 20 µg/ml biotinylated GM1 solution for 1 h. The sensors were then exposed to three concentrations (5, 9, and 12 µM) of WT or the indicated BoNT/DC-H_C_ mutants for 2 min (binding phase), followed by washing with PBS (dissociation phase). The binding parameters were measured and calculated using the Blitz system software (ForteBio).

### Crystallization and structure determination

BoNT/DC-H_C_ in 20 mM HEPES pH 7.0 and 150 mM NaCl, at a concentration of 5 mg/mL, was used for crystallization screening using the sitting drop vapor diffusion method. Before setting up the drops, the protein was incubated with 2.5 mM Sialyl-T for 30 min on ice. Quality crystals appeared after 7 months in 0.2 M ammonium nitrate, 20% PEG3350. The crystals were protected in mother liquor supplemented with 20% glycerol before being flash-frozen in liquid nitrogen. Diffraction data were collected at beamline ×06DA (PXIII), SLS, Switzerland. Data reduction and processing were carried out using XDS and programs from the CCP4 suite. Relevant statistics are shown in Table 1. The structure was solved using molecular replacement, using a previously solved BoNT/DC structure as a model (PDB code: 4ISR, chain A only). The ASU contained two molecules of BoNT/DC-H_C_. Refinement was carried out using Refmac5, interspersed with model building in Coot. The final model was validated using MolProbity.

### Liposome flotation assay

PC was dissolved in chloroform. Gangliosides were dissolved in chloroform:methanol (3:1). PC alone or PC mixed with gangliosides (1%) was dried under nitrogen gas. Lipid films were re-hydrated with the lipid reconstitution buffer (30 mM Tris, 150 mM NaCl, 2 mM MgCl_2_, 2 mM DTT, pH 7.5). Re-suspended lipids were mixed using a shaker at RT for 1 h. Liposomes were generated from re-suspended lipids with an extruder (200 nm pore size, 20 strokes manually, Avanti). Liposomes (75 µl) were incubated with 1 µM proteins (BoNT/DC-H_C_ WT or mutants) in a total volume of 150 µl for 30 min at RT. The liposome–protein mixtures were then added to 100 µl 75% sucrose solution (in lipid reconstitution buffer) to get 250 µl 30% sucrose solution that were loaded as the bottom layer in the centrifuge tube, followed by 200 µl 25% sucrose, and 50 µl lipid reconstitution buffer, as depicted in Fig. 5a. Loaded sucrose gradients were centrifuged at 240,000 × *g* for 1 h (Beckman TLS-55 rotor, OptiMax MAX-XP benchtop centrifuge). After the centrifugation, 50 µl solutions were taken from the top of the centrifuge tube and subjected to immunoblot analysis.

### Data availability

The data and materials that support the findings of this study are available from the corresponding authors upon request. The structure of BoNT/DC-H_C_ complexed with Sialyl-T was deposited in the PDB with the accession code 5LR0.

## Electronic supplementary material

Supplementary Information
