# Supplementary material for: Structural basis for the unique ganglioside and cell membrane recognition mechanism of botulinum neurotoxin DC
Source: Nat Commun. 2017 Nov 21;8:1637. doi: 10.1038/s41467-017-01534-z (PMC5696347; doi:10.1038/s41467-017-01534-z)
Supplement: Supplementary file 1 — Supplementary Information [file 41467_2017_1534_MOESM1_ESM.pdf]

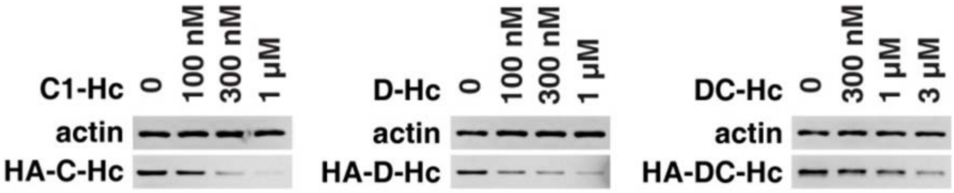

Supplementary Figure 1. Binding sites for BoNT/DC, BoNT/D, and BoNT/C1 on cultured cortical neurons are specific and saturable.

Cultured cortical neurons were exposed to HA-tagged BoNT/C1-H_C (left panel), BoNT/D-H_C (middle panel), or BoNT/DC-H_C (right panel) in the presence of the indicated concentrations of non-tagged H_C proteins, under the same assay conditions described in Fig. 1b-d, except that cell lysates were harvested and subjected to immunoblot analysis. Bound and internalized HA-tagged H_C proteins were detected using an anti-HA antibody. Non-tagged H_C competed with HA-tagged H_C and reduced binding/internalization of HA-tagged H_C proteins, demonstrating that the binding sites for all three toxins are specific and saturable on cultured cortical neurons.

One of two independent experiments is shown.

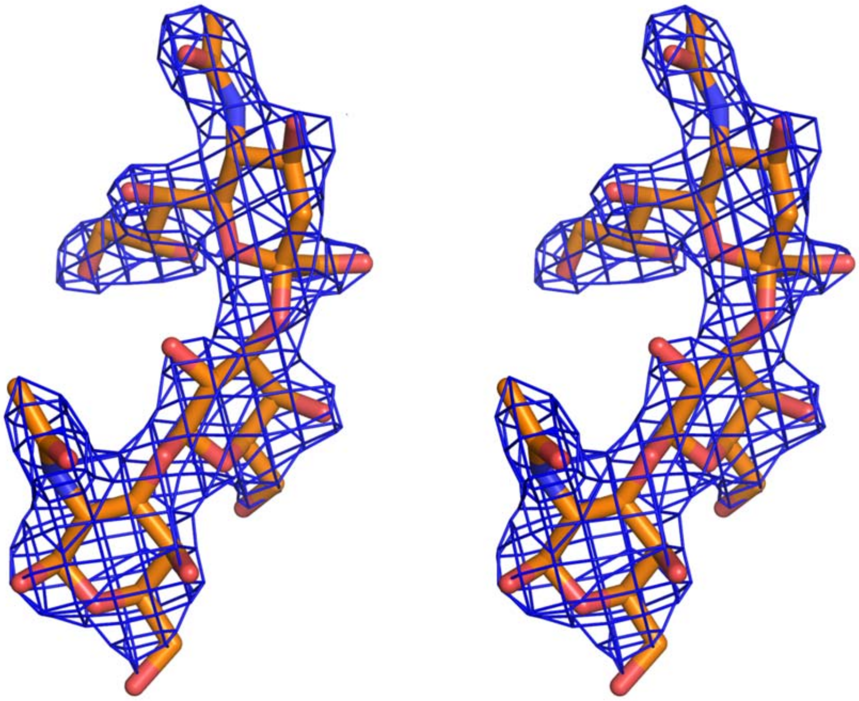

Supplementary Figure 2. Stereo view of Sialyl-T, with its electron density map.
($2F_O$-F_C map contoured at 2.0σ) in blue.

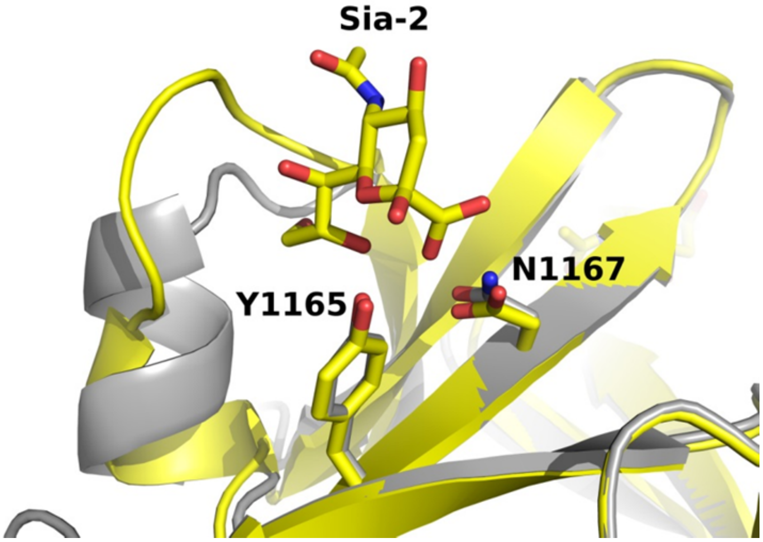

Supplementary Figure 3. The homologous Sia-2 site in BoNT/C1 and BoNT/DC-H_C

Structural superimposition of BoNT/DC (grey) and BoNT/C1 (yellow), highlighting the Sia-2 site with the conserved Y1165/N1167 residues shown as sticks.

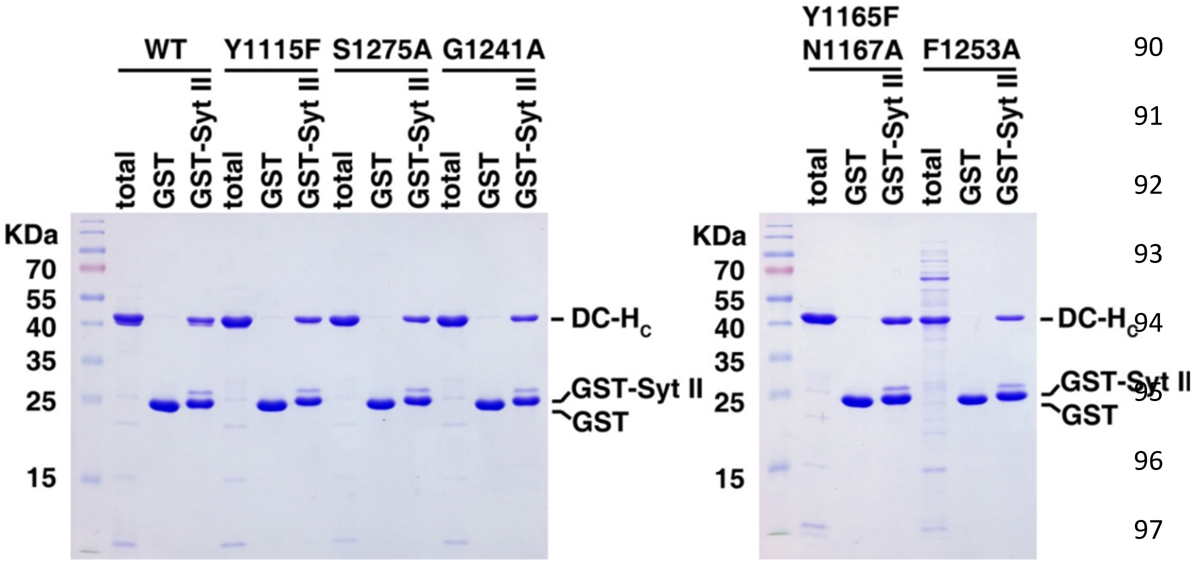

Supplementary Figure 4. Binding of selected BoNT/DC-H$_C$ mutants to recombinant Syt II in pull-down assays.

GST and GST-fused Syt II (40-63) were immobilized on beads (20 µg proteins on 30 µl beads) and incubated with WT or indicated mutants (20 µg) in 200 µl solution. Pellets were analyzed by Coomassie Blue staining. Pull-down of BoNT/DC-H$_C$ mutants and WT BoNT/DC-H$_C$ by Syt II were at similar levels, indicating that these mutants still bind to Syt II and thus are likely folded correctly.

One of two independent experiments is shown.

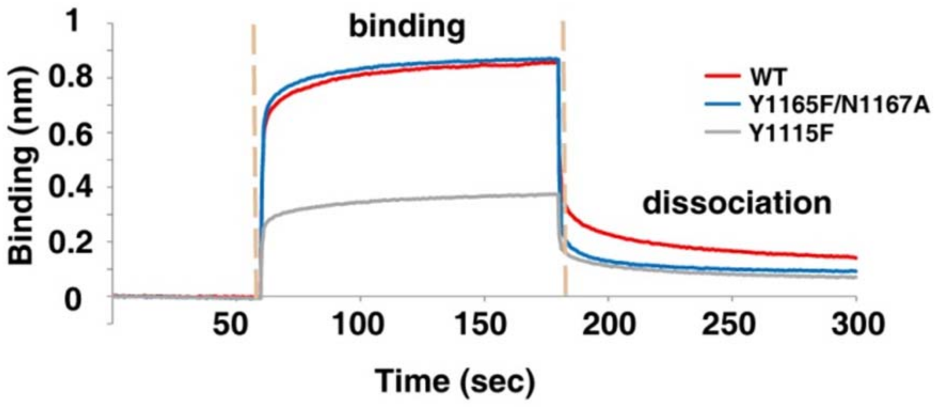

DC-Hc	ka (1/Ms)	ka Error	kd (1/s)	kd Error	K_D (M)
WT	1.32×10^{4}	2.16×10^{3}	3.6×10^{-1}	1.27×10^{-2}	2.76×10^{-5}
Y1165F/N1167A	2.18×10^{4}	5.42×10^{3}	7.75×10^{-1}	3.15×10^{-2}	3.5×10^{-5}
Y1115F	n.d	n.d	n.d	n.d	n.d

Supplementary Figure 5. Characterizing BoNT/DC-H$_C$ binding to biotinylated GM1 with the biolayer interferometry assay.

Biotinylated GM1 was immobilized onto streptavidin-conjugated sensors and subjected to biolayer interferometry assay with the Blitz system (ForteBio). The sensors were exposed to three concentrations (5, 9, and 12 µM) of WT, Y1165F/N1167A, and Y1115F BoNT/DC-H$_C$, followed by washing in PBS. Representative binding and dissociation curves are presented for 12 µM BoNT/DC-H$_C$. The binding parameters were calculated using the Blitz system software (ForteBio). WT and Y1165F/N1167A bind to GM1 with similar dissociation constants (K_D), while Y1115F did not show detectable binding to GM1 under our assay conditions.

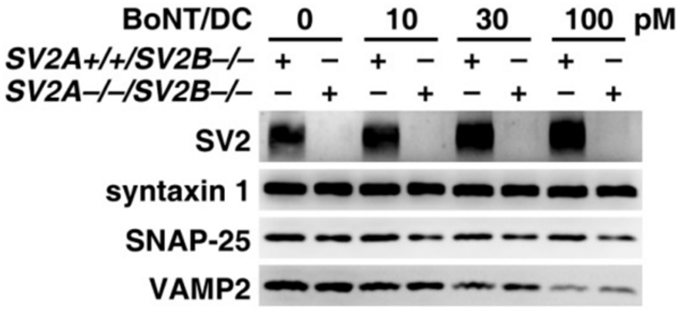

Supplementary Figure 6. Lacking SV2 did not reduce the sensitivity of neurons to BoNT/DC.

Primary cortical neurons express mainly SV2A and SV2B. Thus, cortical neurons cultured from SV2 A/B double-KO mice serve as a SV2-null model. Entry of BoNT/DC into SV2 A/B double-KO neurons were compared to neurons that still express SV2A (*SV2A+/+/SV2B–/–*) under the same assay conditions described in Fig. 1g. Cleavage of VAMP2 was at similar levels in SV2 A/B double-KO neurons and in neurons that express SV2A, suggesting that lack of SV2 did not reduce binding and entry of BoNT/DC.

One of two independent experiments is shown.

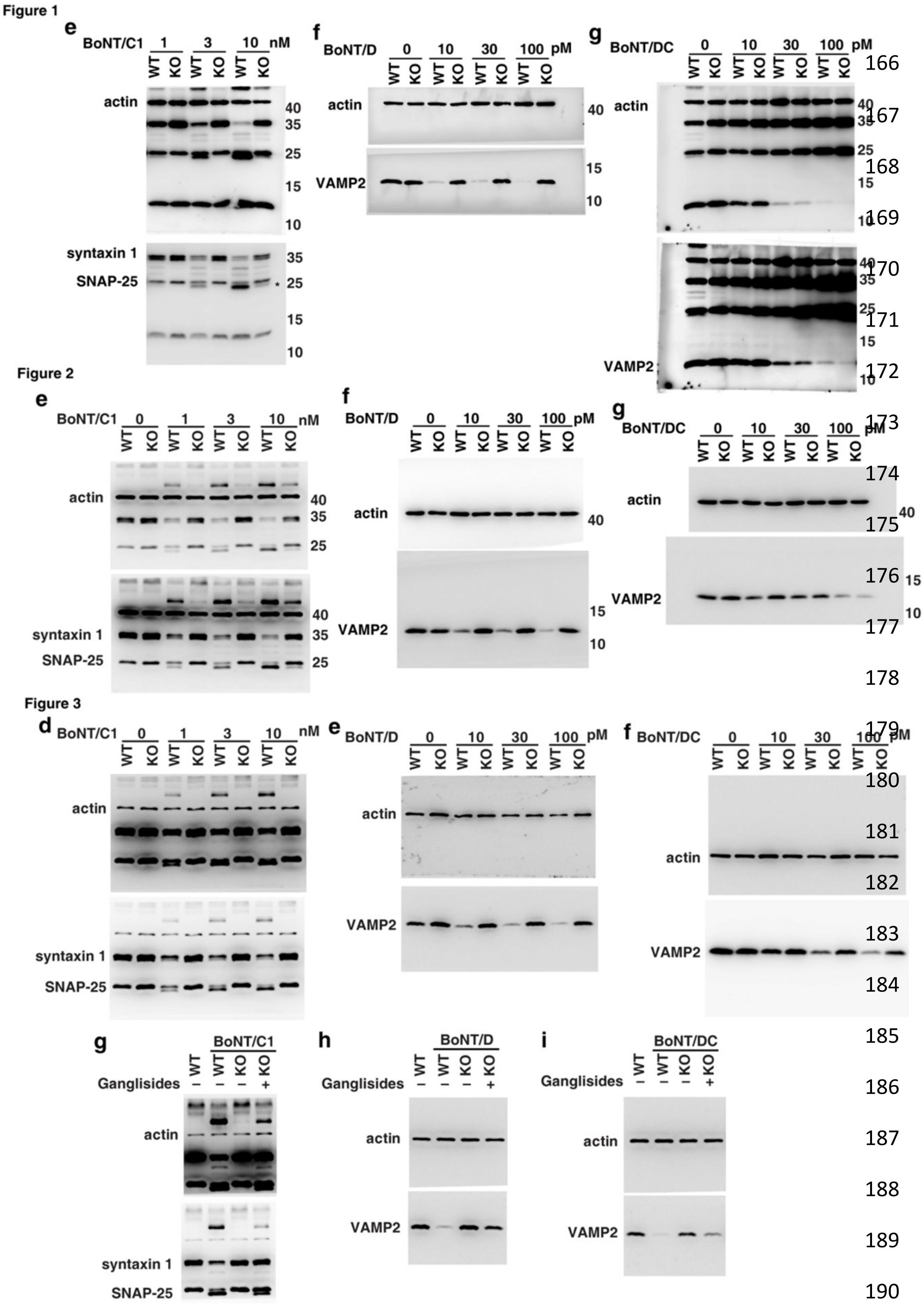

Supplementary Data (Full blot images)

Figure 5

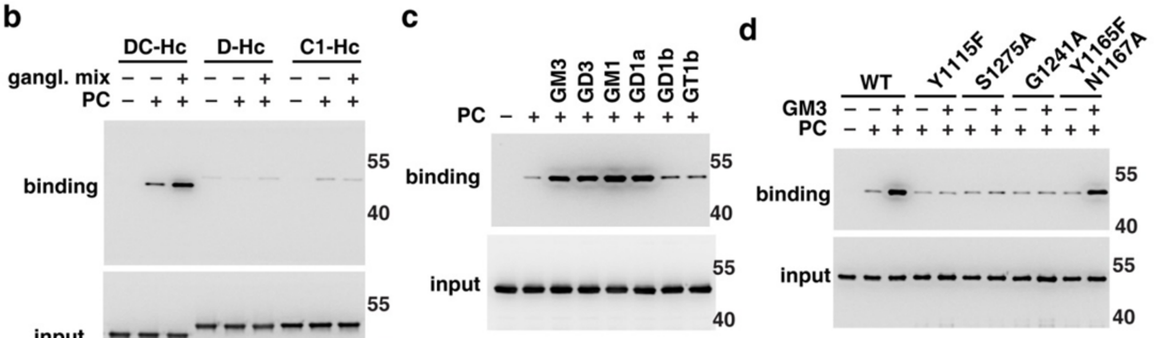

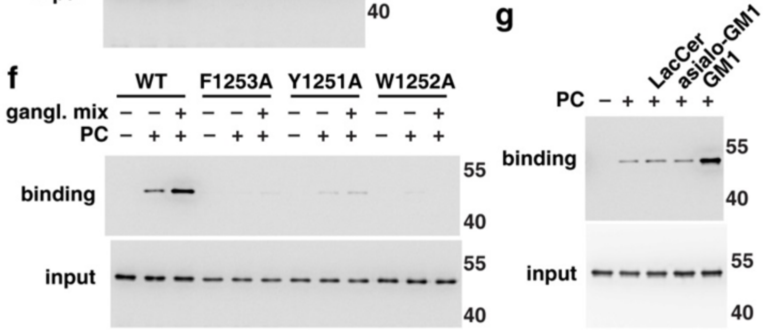

Figure 7

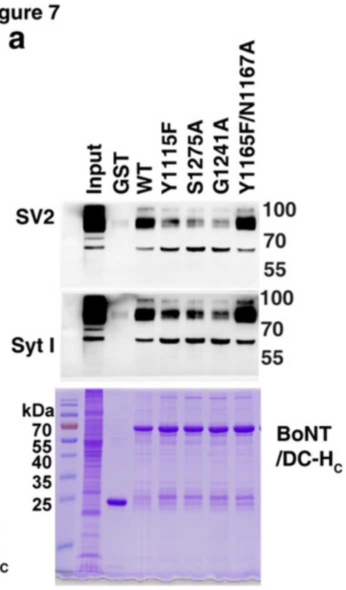

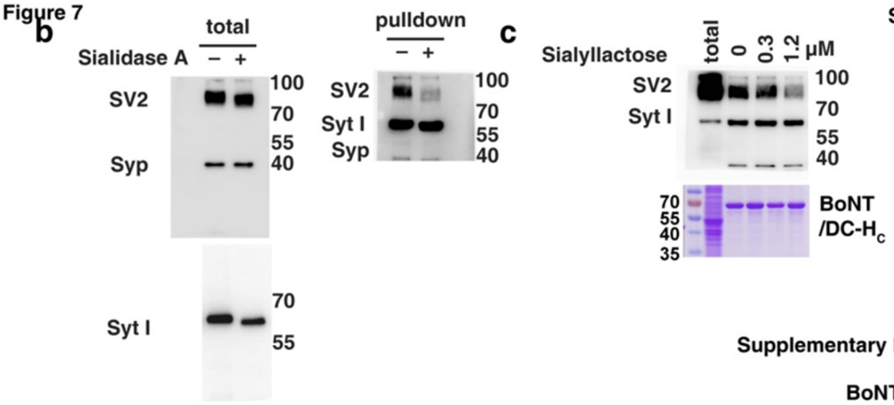

Supplementary Figure 1

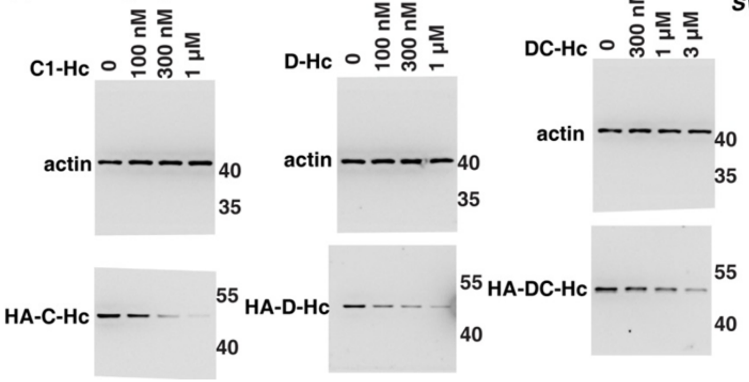

Supplementary Figure 6

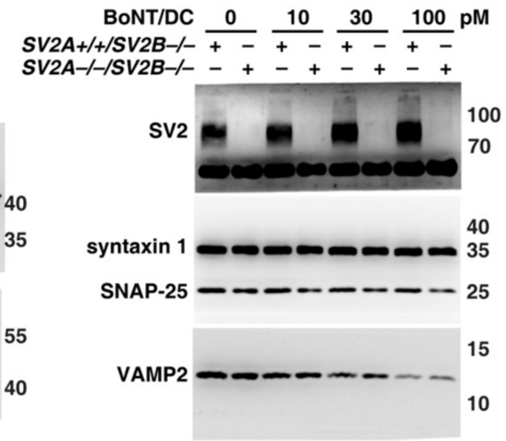

192

193

194 **Supplementary Data (Full blot images)**
